# Infarct tissue heterogeneity by contrast-enhanced MRI is a novel predictor of mortality in patients with coronary artery disease with reduced left ventricular systolic function

**DOI:** 10.1186/1532-429X-13-S1-O7

**Published:** 2011-02-02

**Authors:** Eri Watanabe, Otavio Rizzi Coelho-Filho, François-Pierre Mongeon, Michael Jerosch-Herold, Shuaib M Abdullah, Ron Blankstein, Hiroto Hatabu, Damien Mandry, Bobby Heydari, Rob J van der Geest, Raymond Y Kwong

**Affiliations:** 1 Brigham and Women's Hospital, Boston, MA, USA; 2 Leiden University Medical Center, Leiden, Netherlands

## Introduction

While LVEF is the current most robust marker of mortality in pts with chronic CAD, an improved risk stratification method is necessary for guidance of medical or ICD therapy. Peri-infarct zone (PIZ) as a marker of infarcted heterogeneity has been associated with arrythmogenic substrates and reduced post-MI survival. However, the prognostic implication of PIZ has not been studied in a large cohort of CAD pts with long-term follow-up. In addition, whether this prognostic value of PIZ maintains among different LVEF categories is unknown.

## Purpose

We hypothesized that infarct heterogeneity by CMR provides strong association with patient mortality beyond LVEF.

## Methods

We studied 404 pts with CAD and a LVEF <60% referred for CMR. Ten pts (2%) were excluded for non-CAD pattern of late gadolinium enhancement (LGE). The remaining 394 pts (76% males, mean age 60±12 years, mean LVEF 42±13%) formed the cohort. LGE was performed 10-15 minutes after a total of 0.15-0.2 mmol/kg of Gd-DTPA. A semi-automatic algorithm quantified the infarct core and PIZ using SI criteria of >3SD and >2SD above the remote myocardium, respectively. The PIZ was expressed as a percentage of the total infarct mass (%PIZ). All-cause mortality was obtained by medical record and social security index.

## Results

LGE was present in 297 pts (75%). Mean total infarct mass was 19±23g (14% of LV mass). Mean %PIZ was 19±17%. After a median follow-up of 3.3 years (0.5-8.2 years), 73 (19%) pts died; with 53 deaths occurred among the 304 (77%) pts with a LVEF 30-60%. %PIZ was one of the strongest univariable predictors of mortality (LRχ^2^=12.63, p=0.0004, Table 1) with the hazard increased by 25% per 10% %PIZ increase. Adjusting for age, LVEF, and RVEF, %PIZ maintains a strong association with mortality (HR=1.169, LRχ^2^=4.44, p=0.04). By forward selection, the best overall model for patient mortality included %PIZ, age, LVEF, and history of PCI. In the 304 pts with LVEF>30%, %PIZ demonstrates a robust association with mortality (HR=1.38 per 10% increase in %PIZ, LRχ^2^=17, p<0.0001).

**Table 1:** Variables significantly associated with all-cause mortality by univariable analysis

Parameter	LR chi-square	Hazard ratio	p-value
age	16.2	1.05	<0.0001
LVEF	13.45	0.97	0.0002
%PIZ	12.63	1.25	0.0004
Diabetes	12.55	2.30	0.0004
RVEF	10.89	0.97	0.001
LV end-systolic volume index	7.28	1.01	0.007
History of PCI	6.42	0.49	0.0113
LGE	5.06	2.32	0.0246
LV mass index	4.89	1.01	0.027

## Conclusions

In this large observational cohort with long-term follow-up, infarct heterogeneity measured as %PIZ demonstrated a strong association with patient mortality, especially in pts with a LVEF of 30-60%. This association appears independent of patient age, biventricular systolic function, and LGE presence. Incorporating CMR infarct heterogeneity quantification into the strategic assignment of novel therapy may be warranted in future clinical trials.

